# Standard operating procedure (SOP) for cervical ultrasound cine loop video sequences in the follow-up of differentiated thyroid carcinoma (DTC)

**DOI:** 10.1007/s12020-024-04021-w

**Published:** 2024-09-03

**Authors:** Marc-Patrick Sopuschek, Martin Freesmeyer, Thomas Winkens, Christian Kühnel, Manuela Petersen, Falk Gühne, Anke Werner, Philipp Seifert

**Affiliations:** 1 Clinic of Nuclear Medicine, Jena University Hospital, Jena, Germany; 2 Department of General Visceral Vascular and Transplant Surgery, University Hospital Magdeburg, Magdeburg, Germany

**Keywords:** Ultrasound, Cine Loop, SOP, DTC

## Abstract

**Rationale and objectives:**

Cervical ultrasound (US) is crucial in the follow-up of differentiated thyroid cancer (DTC). However, there are no guidelines for its acquisition and documentation, particularly concerning the role of additional video sequences, known as US cine loops (UCL). The aim of this study is to examine the clinical relevance (CR) of a new Standard Operating Procedure (SOP) for cervical UCL in DTC follow-up.

**Materials and methods:**

A retrospective analysis was conducted on all UCL examinations of DTC follow-up patients at a tertiary care center between January 2010 and February 2018 to determine their clinical significance. The patients were divided into two groups: those with no documented CR (UCL-nCR) and those with documented CR (UCL-CR). The study reviewed the respective written medical US reports that were validated by experienced residents. The UCL-CR were categorized in: confirmation of a suspicious finding that was identified during conventional live US (UCL-CR^con^), identification of a suspicious finding that was not identified during conventional live US (UCL-CR^ide^), and invalidation of a suspicious finding that was identified during conventional live US (UCL-CR^inv^).

**Results:**

A total of 5512 UCLs in 652 DTC patients were analyzed, with 71.5% women and a mean age of 50 years. More than 90% of the tumors were low-risk at initial staging. The mean number of UCLs per patient was 8.5 ± 4.6. Overall, 95 cases of UCL-CR were identified in 82 patients (12.6%), with a patient-based number needed to scan of 8. UCL-CR^inv^ was the most common type of UCL-CR, accounting for 77 (81.1%) of cases. The occurrences of 12 UCL-CR^con^ (12.6%) and 6 UCL-CR^ide^ (6.3%) were correspondingly less frequent. The diagnosis of UCL-CR was confirmed in 91.6% of cases during the clinical course.

**Conclusions:**

In 12.6% of the patients, the additional acquisition and archiving of cervical UCL revealed clinical relevance in the course of DTC disease. The invalidation of suspicious findings through the retrospective analysis of former UCL occurred as the most significant benefit of this method. The UCL SOP can be easily and quickly integrated into the US workflow.

## Introduction

Differentiated thyroid carcinoma (DTC) is one of the most common endocrine carcinomas with an increasing incidence worldwide over the last two decades [1]. This is most likely due to improvements of preoperative diagnostics, particularly of ultrasound sensitivity as a result of major technological developments as well as widespread availability of ultrasound devices [2, 3]. Therefore, smaller tumors and earlier tumor stages are diagnosed, particularly the papillary thyroid microcarcinoma (PTMC) [4]. Consequently, mortality rates did not increase [5]. The main reason for the excellent prognosis of DTC with reported 10-year survival rates of over 95% is the effectiveness of the treatment course including total thyroidectomy (TT) and adjuvant radio iodine therapy (RIT) [6, 7].

However, dependent on the tumor stadium and multiple further intrinsic and extrinsic risk factors, studies revealed tumor recurrence of 5.9-10.3% of the DTC patients within follow-up periods of 54-79 months [8–10]. For papillary thyroid carcinoma (PTC), the peak incidence of recurrence was observed within the first 2 years after TT, mainly diagnosed via ultrasound (US) examinations [10]. Given the increased awareness of the significance to detect clinically indolent tumors and the potential harm and burden associated with overdiagnosis and overtreatment, the approach for treating DTC has recently been critically reassessed [11]. Individual concepts of active surveillance for low-risk DTC with recurrence rates of 1–3% are part of current controversial discussions [12]. However, late tumor relapse can also occur in these tumors and therefore individualized long-term follow-up concepts are recommended [7, 13–15].

The main pillars of regular follow-up examinations are: anamnesis and clinical investigation, thyroid laboratory values (most importantly thyrotropin (TSH), thyroglobulin (TG)) and the investigation of the cervical neck region via ultrasound (US) [7]. In case of suspicion of tumor recurrence, additional extended imaging is regularly conducted, comprising I-131 whole body scintigraphy (WBS) under recombinant human TSH (rhTSH) stimulation and F-18-FDG-PET/CT as well as CT (without contrast agent) and MRI [16, 17].

However, cervical US remains the basic imaging method in DTC treatment. Due to its high soft tissue resolution, a very high sensitivity for the detection of local tumor recurrence and cervical lymph node metastases are reported [18]. However, the quality of the method heavily depends on the examiner’s talent and experience and relatively high intra- and interobserver variability is described for cervical US in general [19]. As there are currently no established procedural recommendations or guidelines for performing and standardizing cervical ultrasound examinations in DTC follow-up, there are potential approaches for improvement.

In general, the acquisition of US video sequences, so called US cine loops (UCLs), has only been implemented in few specialties [20–24]. UCLs are digitally storable in local Picture Archiving and Communication Systems (PACS) and can be retrospectively reevaluated and compared to past investigations. The acquisition of the UCLs can be carried out according to a standard operating procedure (SOP) and thereby be easily reproduced by experts, novices and students. These aspects favor interobserver consistency and are beneficial for medical education.

Due to the potential improvement in diagnostic reliability, particularly in cases with uncertain tumor recurrence, this could be an opportunity to optimize individualized, risk-dependent therapeutic options.

However, the significance of cervical UCLs and specifically for DTC follow-up, lacks clarity. In a recently published study of our research group, the clinical benefit of UCLs for the examination of benign thyroid diseases has been demonstrated [25]. Therefore, the purpose of this study is to introduce a Standard Operating Procedure (SOP) for cervical UCLs and to investigate the value of this methodology in the follow-up of DTC patients. Particular attention was paid to the question of whether the results of the UCL were of clinical relevance (CR) to the patients concerned.

## Materials and methods

### Patients and ethics

All ultrasound examinations of all patients in DTC follow-up (after initial TT and RIT) at a single university nuclear medicine department (uni-centric) between January 2010 and February 2018 were retrospectively analyzed. For patients with UCL-CR, extended follow-up was conducted until September 2020. Exclusion criteria were thyroid carcinomas other than DTC, e.g. medullary thyroid carcinoma, anaplastic thyroid carcinoma, metastases of other carcinomas, lymphoma, since those entities are not treated in nuclear medicine- However, poorly differentiated thyroid carcinomas have not strictly been excluded, because some of those tumors were eligible for RIT and therefore also referred to follow-up examinations in our clinic. The approval of the responsible local ethics committee for the scientific evaluation of the collected data has been obtained (Reference Number: “2023-2967-Daten”).

### Obtained data and cervical US cine loop SOP

The data acquisition was primarily carried out via the digital Radiology Information System (RIS, LORENZO RadCentre, Real-Version 4, iSOFT Health GmbH, Magdeburg, Germany), the local PACS (Centricity Universal Viewer, Version 7.0 Sp1.0.1, GE Healthcare, Chicago, IL, USA), and the clinical information system i.s.h.med (SAP SE GUI, version 7.60 and 7.70 2019/2021; Walldorf, Deutschland). Additionally, examination results from previous analog archive files of the respective patients were considered. The data were documented in Excel (Microsoft Corporation, Version 16.73, Redmond, WA, USA).

**Fig. 1:**
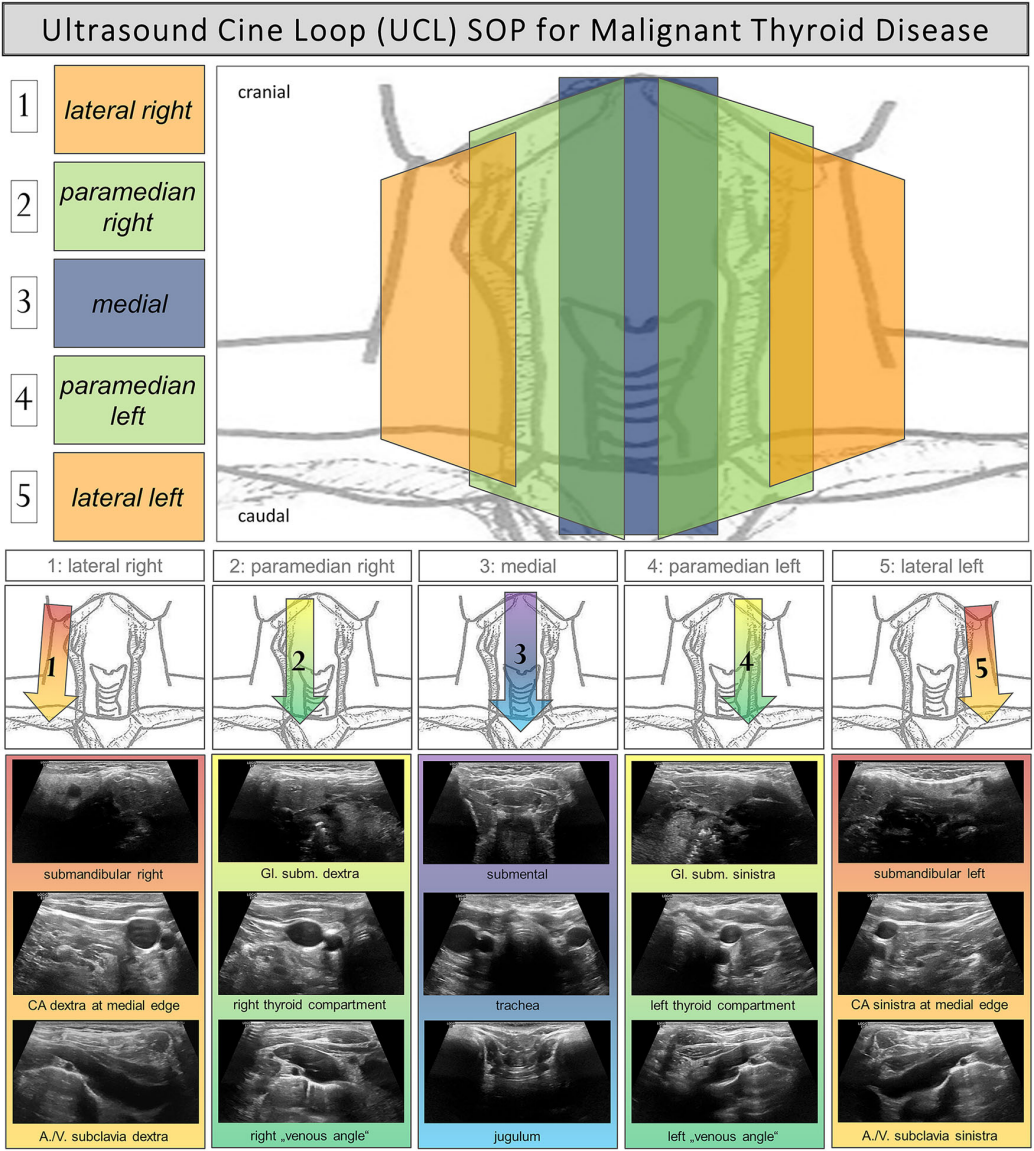
Ultrasound cine loop (UCL) Standard Operating Procedure (SOP) for the follow-up of differentiated thyroid carcinoma (DTC). A total of five transverse cervical UCLs were acquired. [1, 5]: scans of the lateral right/left cervical compartment, extending from submandibular (cranial starting point) to the vasa subclavia (caudal endpoint); [2, 4]: scans of the paramedian cervical compartment including the former thyroid compartments, extending from the submandibular salivary glands (cranial starting point) to the brachiocephalic venous angles (caudal endpoint); [3]: scan of the central cervical compartment, extending from submental (cranial starting point) to the jugulum (caudal endpoint). Gl. subm. Glandula submandibularis, CA carotid artery, A. Arteria, V. Vena

Epidemiological information and patient-specific characteristics, including age, sex, time point of initial diagnosis, follow-up duration, number of US examinations, tumor entity / histopathology, TNM classification and UICC stage (UICC 8th) were documented [26–28]. All findings and assessments of all US examinations were collected. In accordance with the clinical course, ultrasound examinations are conducted at our facility on a periodic basis, with intervals ranging from three to twelve months. The specialty of the investigated patient cohort was the clinically established acquisition of cervical UCLs additionally to the conventional live US of the neck. This was implemented into the clinical routine in order to enable second reading on PACS and for follow-up purposes. All US examiners were asked to record dedicated video sequences of the patients’ necks (cervical UCLs) according to a specific SOP (Fig. 1).

This protocol enabled the investigating physicians to record five short standardized cine loops capturing the entire thyroid compartment as well as the relevant lymph node stations of the ventral and lateral neck regions in transversal orientation. The cine loops were then stored to the local PACS. To avoid blurred images, slow cine loop recording (approximately 10 s favorably containing 200–250 frames per UCL) was recommended.

### US examinations

Both conventional live US and UCL were performed as high-resolution B-mode US. The GE LOGIQ L9 US device (GE Healthcare, Chicago, IL, USA) was used from January 2010 until June 2015 and afterwards the GE LOGIQ E9 US device (GE Healthcare). Both devices were equipped with a linear ML 6–15 probe (GE Healthcare).

Patients were examined in supine position with overflexion of the head. US gel was applied. US examination settings were individually optimized. Usually virtual convex, cross beam as well as contrast harmonic imaging (CHI) were turned on. Further parameters such as frequency (usually between 10 and 15 MHz, standard of 12 MHz), brightness, gain, zoom, and depth were individually adjusted according to the respective findings in order to achieve optimal image quality. The number (usually three) and position of the foci were chosen appropriately.

US examinations were performed by a total of N = 21 different nuclear medicine physicians (containing assistant physicians and residents) with different levels of experience.

### Clinical workflow and scientific analyses of the UCL

Since January 2010 all DTC follow-up US examinations at the Clinic of Nuclear Medicine in the University Hospital Jena are extended by the acquisition of additional UCL by the respective responsible physician. It can be reasonably assumed that these individuals are assistant physicians. A second reading of the UCL is conducted as part of the clinical routine, in comparison to preliminary examinations on the local PACS, by experienced senior physicians. In the event that the UCL yielded clinically relevant results, these were duly included in the medical report. The research component of this study entailed a retrospective evaluation of all medical reports. If a clinical relevance was documented, the respective images were subjected to a review. The retrospective analyses of the clinical data were carried out by one person in correspondence with a local expert.

### Categorization of the UCL findings

The analysis of the UCLs was based on the question of whether they had an impact on the assessment of the US examination and, therefore, a clinical relevance (CR) or not (nCR). CR was identified by clear formulations within the medical reports, i.e. ‘…in retrospective consideration of the previous cine loops…’, ‘…considering former video sequences… ’, ‘…questionable new finding in comparison to older loops…’, etc. Accordingly, four categories were determined:

- UCL-nCR: no documented CR of the UCL
- documented CR of the UCL:
  ○ UCL-CR^con^: confirmation of a suspicious finding identified on conventional live US
  ○ UCL-CR^ide^: identification of a suspicious finding that was not identified on conventional live US
  ○ UCL-CR^inv^: invalidation of a suspicious finding identified on conventional live US

### Extended follow-up of patients with UCL-CR

The follow-up for all patients with UCL-CR was extended until September 2020 in order to verify the diagnosis of the UCL. For these patients, the following further specific data were documented:

- additional extended imaging as a direct consequence of UCL-CR: F18-FDG-PET/CT, I-131 whole-body scintigraphy with rhTSH stimulation (WBS), MRI, CT
- additional interventions as a direct consequence of UCL-CR: surgery, RIT, radiation, systemic therapy

### Data analyses and statistics

Data were recorded in Excel software. Statistical analysis were performed using SPSS software (version 28.0.1.0, IBM Corporation, Armonk, New York, USA). Graphics, Figures and charts were created in PowerPoint software (version 16.74, Microsoft Corporation, Redmond, Washington, USA). For the comparison of not normally distributed metric values, Mann–Whitney *U* test (MWU) was used. For the comparison of nominal and ordinal values, Chi-Square test (χ2) was used. *P-values* < 0.05 were considered statistically significant.

## Results

**Fig. 2:**
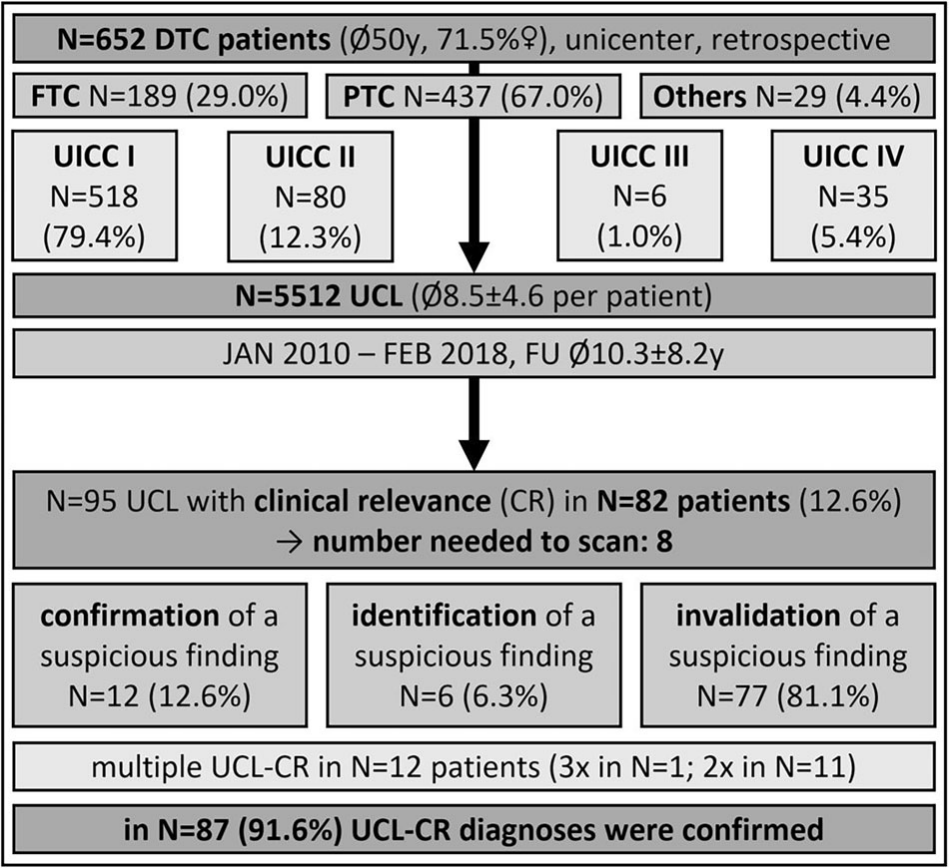
Flowchart of the study methods and results. N number, DTC differentiated thyroid carcinoma, SD standard deviation, PTC papillary thyroid carcinoma, FTC follicular thyroid carcinoma, UICC Union of International Cancer Control 8th version (2017), FU follow-up, UCL standardized cervical ultrasound cine loops, UCL-CR standardized cervical ultrasound cine loops with clinical relevance

A total of 652 patients and 5512 UCLs were included. On average, a single UCL took about one minute per patient following the introduced SOP. Two thirds of the patients suffered from a papillary thyroid carcinoma (PTC). Mainly early tumor stages UICC I and UICC II (UICC 8th) were diagnosed [28]. During the observation period each patient underwent an average of approximately eight follow-up investigations. Individual follow-up length after initial diagnosis and patients’ age were subject to a large dispersion. Overall, 95 UCL-CR in 82 patients (12.6%) were identified (number of patients needed to scan: N = 8), mainly UCL-CR^inv^ (81.1%). In N = 12 patients, multiple UCL-CR were identified during the follow-up and treatment course. The diagnosis of UCL-CR was confirmed in 91.6% of cases during the clinical course. An overview of the study design and the main results are shown in the graphical flowchart Fig. 2.

Firstly, the entire patient cohort was taken into account (patient-based data). A comparison of patient and DTC characteristics between UCL-CR and UCL-nCR patients was carried out. No significant differences regarding sex, age, time of follow-up, tumor entity, N-staging, M-staging, and UICC were found. UCL-CR patients received significantly higher numbers of UCL (p < 0.001) and showed higher T-stagings (p = 0.011), particularly T3. Detailed results are shown in Table. 1.

**Table. 1:** Patient and DTC characteristics, comparison between UCL-CR and UCL-nCR patients

Parameter		All patients (N = 652)	UCL-CR (N = 82)	UCL-nCR (N = 570)	*p*-values
		N (%) / mean ± SD (Median, Min-Max)			
Sex	female	466 (71.5)	60 (73.2)	406 (71.2)	0.716
	male	186 (28.5)	22 (26.8)	164 (28.8)	
Age at ID [years]		50.0 ± 15.6 (50, 8–89)	51.6 ± 14.7 (51, 16–89)	49.8 ± 15.7 (50, 8–89)	0.451
FU since ID [years]		10.3 ± 8.2 (9, 0–44)	11.1 ± 7.5 (10, 1–39)	10.2 ± 8.2 (9, 0–44)	0.155
Number of UCL		8.5 ± 4.6 (8, 1–28)	11.7 ± 4.6 (11, 3–28)	8.0 ± 4.5 (8, 1–27)	<0.001
Entity	*PTC*	434 (66.6)	48 (58.5)	386 (67.7)	0.247
	*FTC*	189 (29.0)	29 (35.4)	160 (28.1)	
	*other DTC*	29 (4.4)	5 (6.1)	24 (4.2)	
T staging	*T1*	243 (37.7)	20 (24.4)	223 (39.1)	0.011
	*T2*	179 (27.5)	25 (30.5)	154 (27.0)	
	*T3*	128 (19.6)	25 (30.5)	103 (18.1)	
	*T4*	29 (4.4)	6 (7.3)	23 (4.0)	0.232
	*Tx*	73 (11.2)	6 (7.3)	67 (11.8)	
	*multiple*	54 (8.3)	4 (4.9)	50 (8.8)	
N staging	*cN0*	432 (66.3)	49 (59.8)	383 (67.2)	0.310
	*pN0*	119 (18.3)	16 (19.5)	103 (18.1)	
	*N*+	101 (15.5)	17 (20.7)	84 (14.7)	
M staging	*M0*	622 (95.4)	77 (93.9)	545 (95.6)	0.489
	*M*+	30 (4.6)	5 (6.1)	25 (4.4)	
UICC 8th	*I*	518 (79.4)	60 (73.2)	458 (80.4)	0.315
	*II*	80 (12.3)	13 (15.9)	67 (11.8)	
	*III*	6 (1.0)	2 (2.4)	4 (0.7)	
	*IV*	35 (5.4)	6 (7.3)	29 (5.1)	
	*X*^a^	13 (2.0)	1 (1.2)	12 (2.1)	

*UCL-CR* standardized cervical ultrasound cine loops with clinical relevance, *UCL-nCR* standardized cervical ultrasound cine loops without clinical relevance, *N* number, *SD* standard deviation, *ID* initial diagnosis, *FU* follow-up, *PTC* papillary thyroid carcinoma, *FTC* follicular thyroid carcinoma, *DTC* differentiated thyroid carcinoma, *UICC* Union of International Cancer Control 8th version (2017)

^a^UICC X contains patients > 55 years without known T-staging

Secondly, only UCL-CR patients (N = 82) were evaluated in further detail. In 12 of the 82 patients multiple UCL-CR were recorded within the course of the DTC follow-up. Therefore, we identified a total of 95 UCL-CR examinations. In one patient, 3 UCL-CR were detected (3 times UCL-CR^inv^). In 11 patients, UCL-CR were identified twice (8 patients with UCL-CR^inv^ twice; 2 patients with UCL-CR^inv^ + UCL-CR^ind^; 1 patient with UCL-CR^con^ + UCL-CR^inv^). Therefore, *Part II* results are case based (not patient-based), detailed results are shown in Table. 2. Due to the uneven distribution between the UCL-CR categories and the overall relatively low number of UCL-CR in general (UCL-CR^con^: N = 12, UCL-CR^ide^: N = 6, UCL-CR^inv^: N = 77), no analyses of distribution differences between the groups were conducted. However, the results suggest that UCL-CR^con^ patients exhibited no discernible difference in UICC stages distribution when compared to the UCL-CR^ide^ and UCL-CR^inv^ patients. For UCL-CR^con^ patients, the initial risk appears to be higher, the initial response worse, and the thyroglobulin levels higher at the time point of UCL-CR.

**Table. 2:** Clinical data of UCL-CR patients (N = 95 UCL-CR in 82 patients) in relation to the UCL-CR category

Parameter		UCL-CR^con^ (N = 12)	UCL-CR^ide^ (N = 6)	UCL-CR^inv^ (N = 77)
		N (%) / mean ± SD (Median, Min-Max)		
No. of patients		11	6	65
UICC 8th	*I*	7 (63.6)	3 (50.0)	51 (78.5)
	*II*	2 (18.2)	2 (33.3)	9 (13.8)
	*III*	0 (0.0)	0 (0.0)	2 (3.1)
	*IV*	2 (18.2)	1 (16.7)	3 (4.6)
ATA risk stratification (7)	*low*	1 (8.3)	3 (50.0)	44 (57.1)
	*intermediate*	5 (41.7)	1 (16.7)	17 (22.1)
	*high*	6 (50.0)	2 (33.3)	16 (20.8)
ATA initial response (7)	excellent	5 (41.7)	3 (50.0)	47 (61.0)
	biochemical incomplete	1 (8.3)	1 (16.7)	10 (13.0)
	structural incomplete	6 (50.0)	2 (33.3)	14 (18.2)
	indeterminate	0 (0.0)	0 (0.0)	6 (7.8)
Thyroglobulin at UCL-CR, unstimulated	*<0.8* *ng/ml*^a^	6 (50.0)	4 (66.7)	56 (72.7)
	*0.8–1.0* *ng/ml*	0 (0.0)	1 (16.7)	2 (2.6)
	*1.1–10.0* *ng/ml*	2 (16.7)	1 (16.7)	5 (6.5)
	> *10.0* *ng/ml*	4 (33.3)	0 (0.0)	14 (18.2)
ID until UCL-CR [month]		78.3 ± 122.8 (30, 6–449)	107.8 ± 79.3 (79, 38–242)	117.3 ± 83.5 (99, 4–368)
extended imaging as direct consequence of UCL-CR result		in 10 patients (83.3)	in 5 patients (83.3)	no patients (0.0)
*F-18-FDG PET/CT*		10 (83.3)	3 (50.0)	0 (0.0)
*I-131 WBS (rhTSH stimulation)*		10 (83.3)	5 (83.3)	0 (0.0)
*MRI*		1 (8.3)	0 (0.0)	0 (0.0)
*CT*		1 (8.3)	2 (33.3)	0 (0.0)
*none*		2 (16.7)	1 (16.7)	77 (100.0)
intervention as direct consequence of UCL-CR result		in 7 patients (58.3)	no patients (0.0)	no patients (0.0)
*surgery*		7 (58.3)	0 (0.0)	0 (0.0)
*RIT*		6 (50.0)	0 (0.0)	0 (0.0)
*radiation*		0 (0.0)	0 (0.0)	0 (0.0)
*systemic therapy*		0 (0.0)	0 (0.0)	0 (0.0)
*none*		5 (41.7)	6 (100.0)	77 (100.0)
FU after UCL-CR [month]		59.5 ± 29.9 (53, 33–119)	71.0 ± 27.5 (64, 43–115)	63.8 ± 22.4 (57, 31–121)
UCL-CR resultconfirmed in further treatment course		validation of malignancy (in 10 patients)	validation of malignancy (in 1 patient)	no malignancy diagnosed (in 76 patients)
*only standard FU*		1 (8.3)	0 (0.0)	51 (66.2)
*additional extended imaging*		9 (75.0)	1 (16.7)	25 (32.5)
*histopathological*		7 (58.3)	0 (0.0)	0 (0.0)
UCL-CR result not confirmed in further treatment course		no malignancy diagnosed in 2 patients (16.7)	no malignancy diagnosed in 5 patients (83.3)	malignancy diagnosed in 1 patient (1.3)^b^
*only standard FU*		1 (8.3)	1 (16.7)	0 (0.0)
*additional extended imaging*		1 (8.3)	4 (66.7)	1 (1.3)
*histopathological*		0 (0.0)	0 (0.0)	1 (1.3)

*UICC* Union of International Cancer Control 8th version (2017), *ATA* American Thyroid Association, *UCL-CRcon* standardized cervical ultrasound cine loop with clinical relevance—confirmation of a suspicious finding identified on conventional live ultrasound, *UCL-CRide* identification of a suspicious finding that was not identified on conventional live ultrasound, *UCL-CRinv* invalidation of a suspicious finding identified on conventional live ultrasound, *N* number, *SD* standard deviation, *ID* initial diagnosis, *FU* follow-up, *F-18-FDG* fluorine-18-fluorodeoxyglucose, *PET/CT* positron emission tomography computed tomography, *I-131* iodine-131, *WBS* whole body scintigraphy, *MRI* magnetic resonance imaging, *CT* computed tomography, *RIT* radio iodine therapy, *TSH* thyrotropin

^a^Sensitivity of the local laboratory test during the study period

^b^*Case* is shown in Fig. 6

**Fig. 3:**
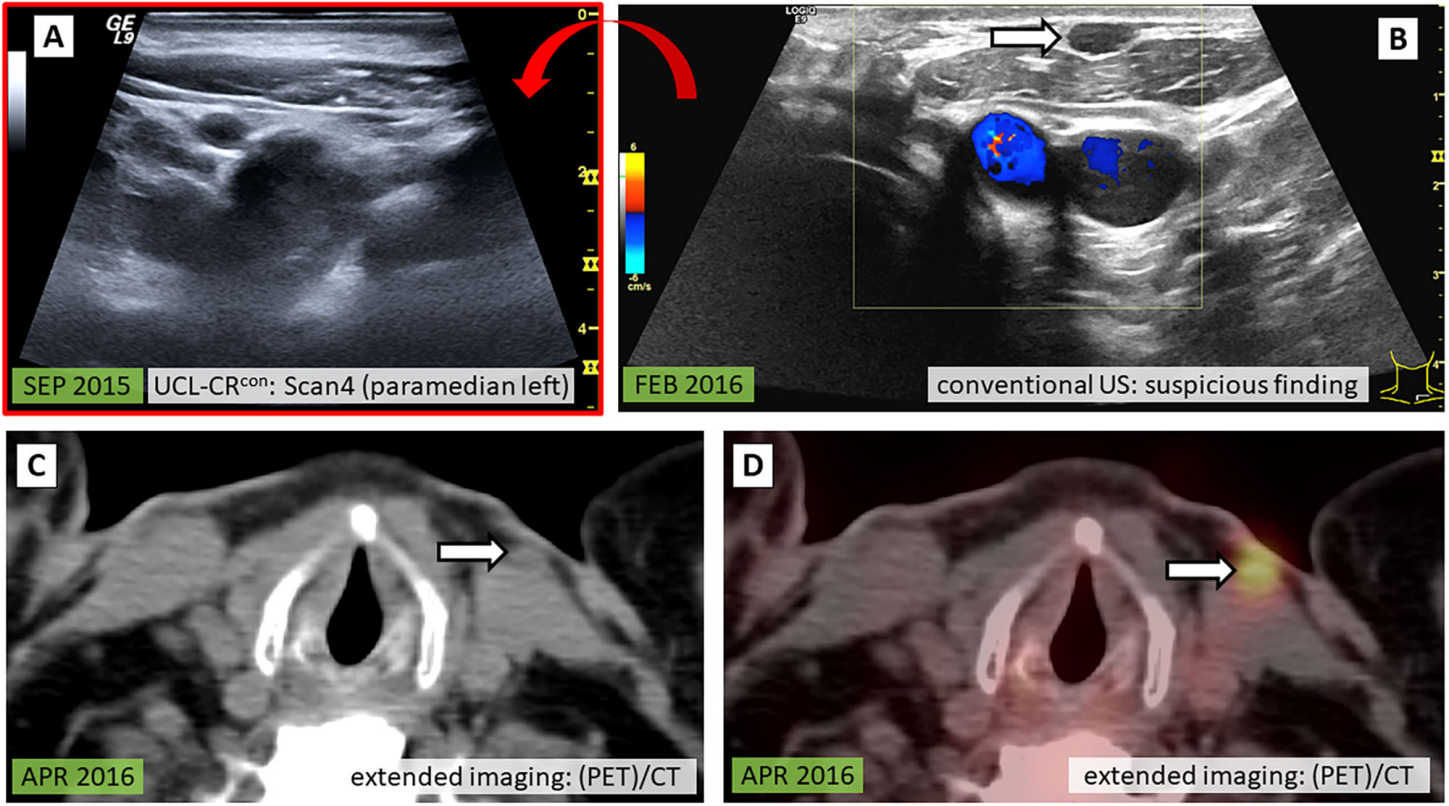
Patient case I: UCL-CR^con^ with confirmed diagnosis. 54-year-old male at initial diagnosis 2008 (FTC pT3 cN1 cM0). Conventional US revealed a suspicious subcutaneous nodule measuring 5 mm [**B**, white arrow] along with increasing TG levels (SEP 2015: 14.8 ng/ml, FEB 2016: 35.1 ng/mL, unstimulated). The retrospective PACS review of the UCL from SEP 2015 [**A**] confirmed that the identified lesion is new and thus highly suspicious for a cervical FTC relapse. Consequently, additional extended imaging was conducted. The nodule was reproduced on CT [**C**, white arrow] and showed elevated FDG uptake on PET/CT [**D**, white arrow]. The subsequent surgical removal confirmed a late relapse of FTC. UCL-CR^con^ standardized cervical ultrasound cine loops with clinical relevance - confirmation of a suspicious finding seen on conventional live ultrasound, US ultrasound, PET/CT positron emission tomography computed tomography, FTC follicular thyroid carcinoma, TG thyroglobulin

**Fig. 4:**
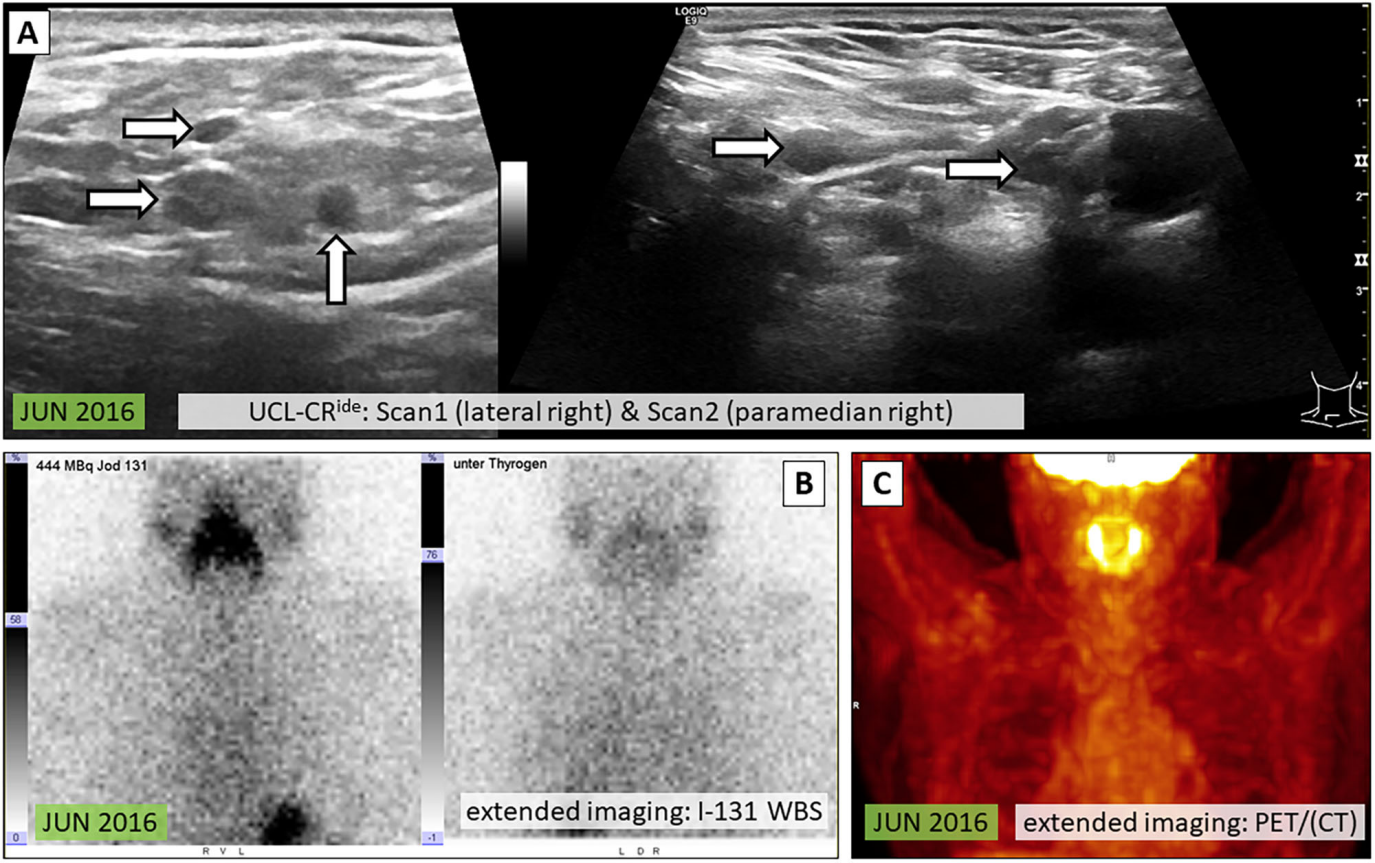
Patient case II: UCL-CR^ide^ without confirmed diagnosis / false diagnosis. 64-year-old female at initial diagnosis 2008 (PTC pT1b pN1b cM0). The PACS review of the UCL revealed multiple suspicious lymph nodes supraclavicular right [**A**, white arrows], measuring up to 7 mm, that have not been identified on conventional live US during regular FU. TG was not measurable. Additional extended imaging included I-131 WBS [**B**] and F-18-FDG PET/CT [**C**] did not show any suspicious findings. No PTC relapse occurred in further FU. Thus, the diagnosis of the UCL-CR could not be confirmed. UCL-CR^ide^ standardized cervical ultrasound cine loops with clinical relevance—identification of a suspicious finding that was not identified on conventional live ultrasound, PTC papillary thyroid carcinoma, PACS Picture Archiving and Communication System, US ultrasound, FU follow-up, TG thyroglobulin, I-131 WBS iodine-131 whole body scintigraphy, F-18-FDG PET/CT fluorine-18-fluorodeoxyglucose positron emission tomography computed tomography

**Fig. 5:**
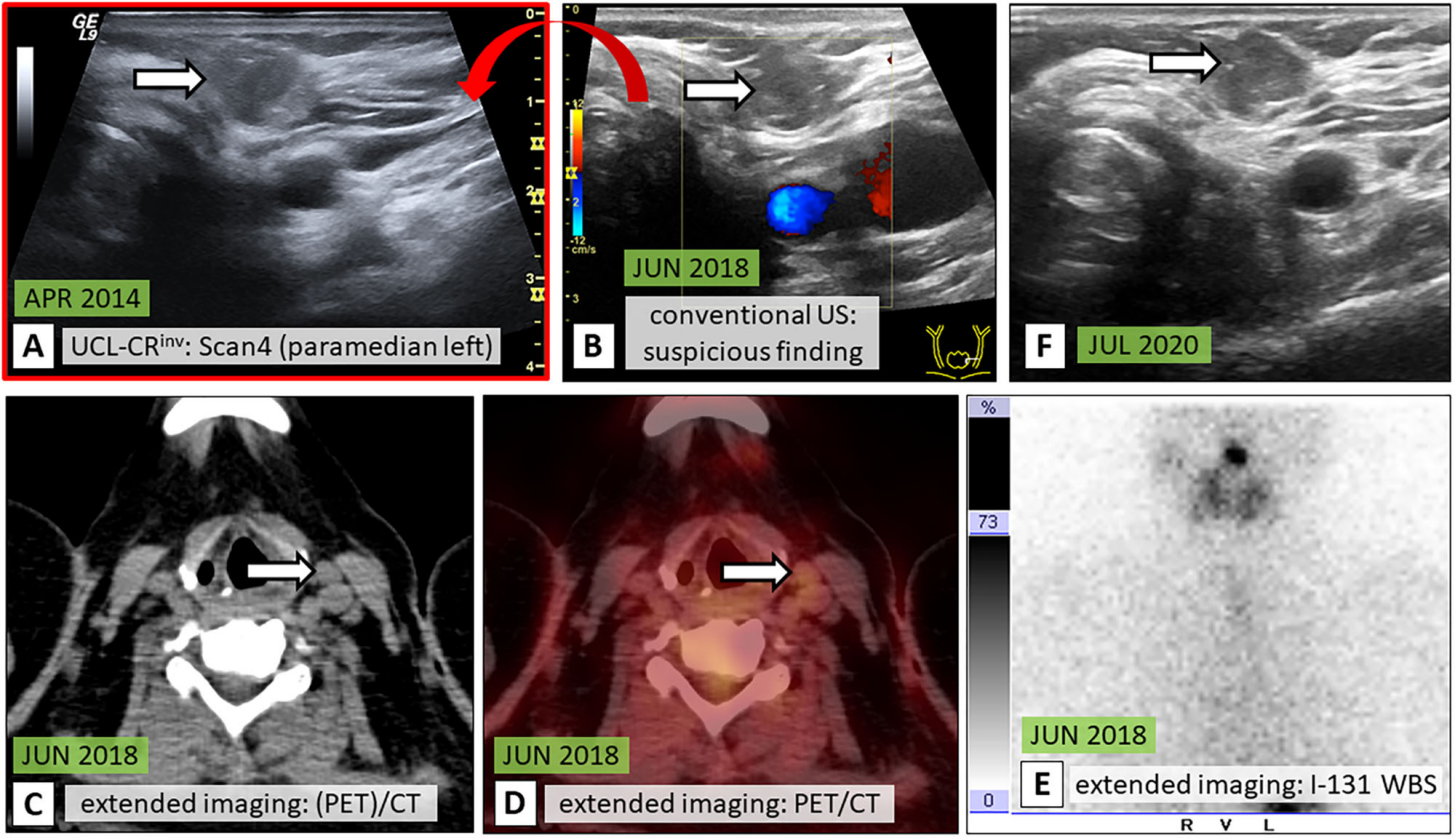
Patient case III: UCL-CR^inv^ with confirmed diagnosis. 49-year-old female at initial diagnosis 2010 (PTC pT2m pN0 cM0). Conventional US revealed a suspicious nodule in the former left-sided thyroid lodge, measuring 9 mm [**B**, white arrow] after 8 years of FU (JUN 2018). The retrospective PACS review of the UCL from APR 2014 confirmed that the identified lesion is constant over more than 4 years [**A**, white arrow]. Nevertheless, additional extended imaging was conducted. The lesion was visible on CT [**C**, white arrow] and did neither show pathological FDG uptake on F-18-FDG PET/CT [**D**, white arrow] nor pathological I-131 uptake on WBS [**E**]. The nodule was constant on long-term US FU [**F**, white arrow]. TG levels were negative throughout the entire time period. UCL-CR^inv^ standardized cervical ultrasound cine loops with clinical relevance—invalidation of a suspicious finding identified on conventional live US, PTC papillary thyroid carcinoma, US ultrasound, FU follow-up, PACS Picture Archiving and Communication System, I-131 WBS iodine-131 whole body scintigraphy, F-18-FDG PET/CT fluorine-18-fluorodeoxyglucose positron emission tomography computed tomography, TG thyroglobulin

**Fig. 6:**
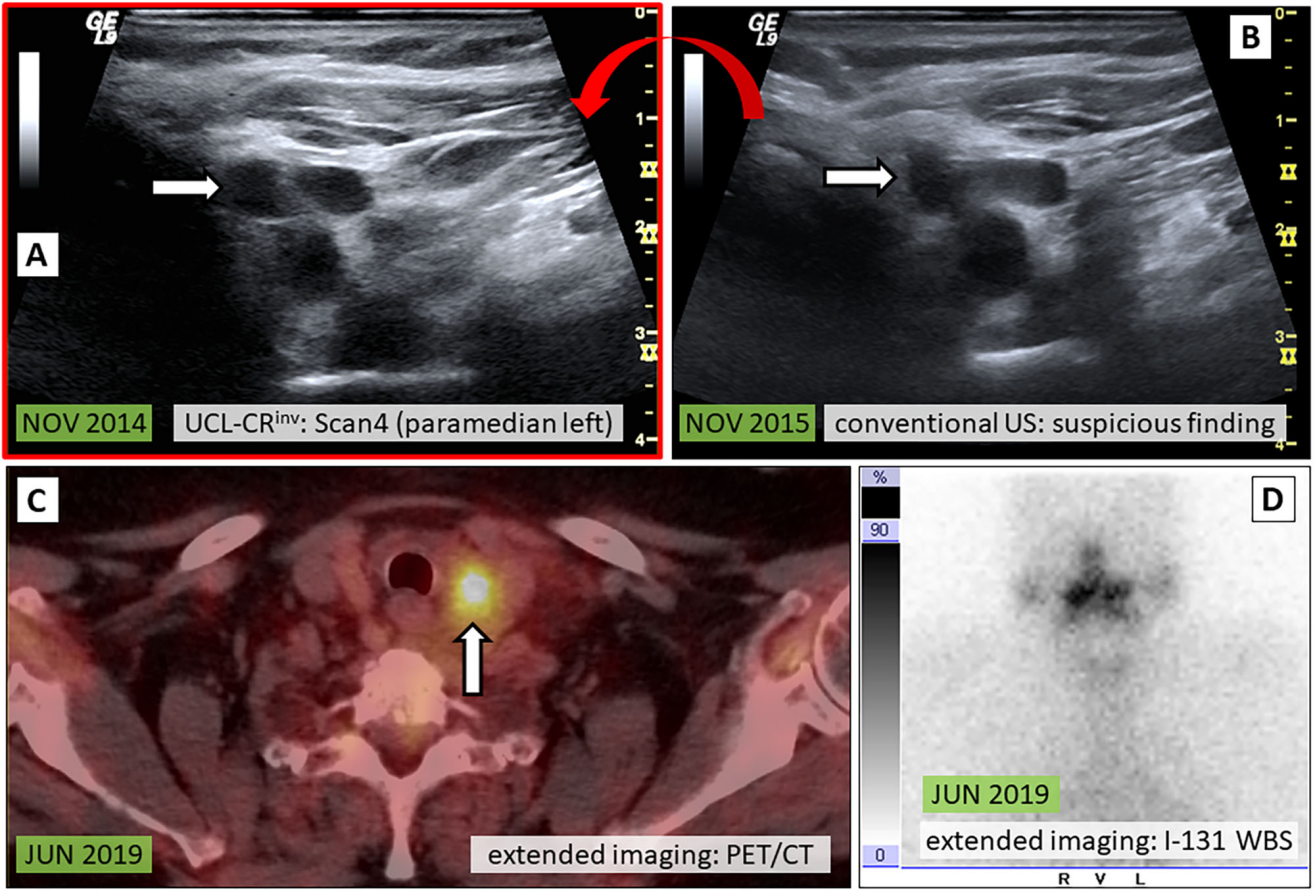
Patient case IV: UCL-CR^inv^ without confirmed diagnosis / false diagnosis. 56-year-old female at initial diagnosis 1999 (PTC pT2 cN0 cM0). Conventional US revealed a suspicious nodule in the former left-sided thyroid lodge, measuring 12 mm [**B**, white arrow] after 6 years (NOV 2015). The retrospective PACS review of the UCL from NOV 2014 revealed the same lesion without significant increase in size [**A**, white arrow], which was consequently assessed as benign (scar). However, TG levels were slowly rising throughout the following years and finally additional extended imaging was conducted. A pathological FDG uptake was seen on F-18-FDG PET/CT [**C**, white arrow], but I-131 WBS did not show iodine uptake [**D**, white arrow]. Resection of the lesion confirmed a local tumor relapse of the PTC. UCL-CR^inv^ standardized cervical ultrasound cine loops with clinical relevance - invalidation of a suspicious finding identified on conventional live US, PTC papillary thyroid carcinoma, US ultrasound, PACS Picture Archiving and Communication System, TG thyroglobulin, F-18-FDG PET/CT fluorine-18-fluorodeoxyglucose positron emission tomography computed tomography, I-131 WBS iodine-131 whole body scintigraphy

In the following (Figs. 3–6), typical UCL-CR situations are explained by 4 patient case examples.

## Discussion

Cervical US is a common clinical practice and a fundamental component of DTC follow-up care, particularly for the identification of loco-regional tumor recurrence. However, a major disadvantage of the methodology is the examiner dependency. Even today, most US results are only documented by static image captures in clinical routine, making a comprehensive second reading impossible and therefore leading to the possibility of overlooked relevant findings [20]. In other medical specialties’, recordings of US video sequences, also known as UCL, are an established part of the workflow [29–31].

In almost all other tomographic examinations, such as CT or MRI, the documentation of the scan area in layers is a naturally standard procedure. It is therefore logical and appropriate to apply the same approach to US. During the US examination, the cervical compartments are scanned several times. Consequently, storing these natural movements in a UCL requires minimal additional effort. Capturing cervical UCL allows comprehensive morphological information to be archived in the PACS, which is particularly useful for second reading by experienced colleagues, long-term follow-up assessments and teaching to novices and students. Furthermore, a clearly structured and easy to apply SOP may enable non-physician personnel to perform the examination. This was demonstrated in a previous study using UCL in benign thyroid diseases. The UCL images acquired by medical technical assistants allowed for a complete and reliable structured report based solely on PACS video data [25, 32].

The SOP presented in this study is easy to apply and can be carried out in less than one minute. It is possible to capture the entire para-tracheal lodge (former thyroid gland bed) and its surroundings including all cervical lymph node regions via five short standardized transverse video sequences and save them to the local PACS. We have been using this method for over ten years and the subjective benefits go far beyond the scientifically reported results of this work. Partially, older UCLs are already viewed in the PACS before a current US examination, allowing for the identification of questionable findings and the formulation of a valid statement to the patient immediately after the examination. Such a procedure is not always documented in the written reports as a benefit of the UCL. Consequently, it can be postulated that the influence of the UCL on clinical treatment may be even greater than the scientific evaluation of the data from this study suggests.

In the context of long-term courses of DTC with an overall excellent prognosis, but with the necessity for lifelong follow-up due to the risk of late-onset tumor recurrence, the use of standardized UCL is particularly advantageous. DTCs, which are well-differentiated tumors, usually grow slowly and the main area of recurrences is the neck – an area that is very accessible to US diagnostics. By continuous UCL recordings over the long follow-up period, slow-growing tumors can be differentiated from benign findings of constant size. Nevertheless, the utility of frequent ultrasound examinations for low and intermediate risk PTC is a topic of debate in the literature, particularly in cases where there is an excellent therapy response and no evidence of disease [33–35]. The suggestion is to limit the number of serial neck US examinations in order to reduce the likelihood of false positive findings and overdiagnosis.The implementation of UCL additional to conventional cervical US into the clinical follow-up routine of DTC patients resulted in 95 cases with clinical benefits (UCL-CR) within 82 patients (12.6%). This means a number needed to scan of N = 8 (patient-based). The largest proportion (81.1%) of UCL-CR cases were the retrospective invalidation (UCL-CR^inv^) of initially suspicious abnormalities during conventional live US. This fact is of particular value from an economic perspective. Suspicious US findings in DTC patients are typically clarified by timely follow-ups, which require the patient to return to the clinic, or complex and cost-intensive supplementary examinations, including FDG-PET/CT, an I-131-WBS under rhTSH stimulation, MRI, CT, and their combination. The invalidation of suspicious findings of the conventional US by usage of UCL is therefore of significant benefit to patients and the healthcare system. On the other hand, in seven patients of the UCL-CR^con^ and UCL-CR^ide^ group, suspicious US findings of UCL could not be confirmed and the possibility of false diagnosis and following consequences due to UCL must be acknowledged.

The patient cohort of this study did not undergo preselection and can be regarded as representative for a region with a long history of iodine deficiency [36–38]. In the comparison of UCL-CR and UCL-nCR patients no relevant differences in the distribution of sex, age, entity, tumor stage or follow-up period were identified. A higher number of UCL per patient was seen in the UCL-CR group, which is caused by more frequently implemented re-examinations due to clinically relevant US findings.

The higher T-stages in the UCL-CR group may be related to overall higher recurrence risks in advanced tumor stages, but otherwise mostly UCL-CR invalidations were documented. Therefore, it can be assumed to be a coincidence. Patients with UCL-CR had an overall higher number of UCL in the course of follow-up presumably because higher-frequency US examinations were carried out as a consequence of the clinical relevance of the UCL.

Overall, 33 (40.2%) patients with UCL-CR underwent additional extended imaging. For UCL-CR^con^ and UCL-CR^ide^, this is mandatory to validate suspicious findings and plan further treatment. In contrast, all UCL-CR^inv^ patients who received additional extended imaging did so due to other indications, such as elevated TG, other suspicious findings (not related to DTC) on imaging or clinical examinations, and other diseases.

In total, 87 (91.6%) case-based and 74 (90.2%) patient-based UCL diagnoses were confirmed as correct. This represents a relatively high precision rate, favorable negative predictive values, increasing significance, and clinical expressiveness of the US-based examination in the standard DTC follow-up. Verification was based on follow-up investigations, additional extended imaging and clinical examinations, laboratory values (TG course), and histopathological analyses.

### Limitations

With regard to the study design, several limitations must be admitted that may have influenced the results and their interpretation:

- The true rate of UCL-CR is empirically higher. In the current study, only written reports were considered, thereby identifying only cases with documented UCL benefit. It is possible that not all cases with clinical relevance have been perfectly documented in the clinical routine.
- The stored UCL were and continue to be clinically retrieved in all cases. However, only if the respective UCL yielded clinically relevant information in addition to that obtained from the live US was this added value documented in the medical reports. This approach is susceptible to the potential for methodological inconsistencies, particularly in regard to interobserver variability. One way to improve this in future research is the usage of structured reporting that has been shown to significantly improve the quality of US investigations in DTC FU [39, 40].
- The image quality of the acquired video sequences is most often below that of static image captures. Reasons are patient intrinsic factors i.e., movement during acquisition and shortness of breath, artifacts due to insufficient US gel application and too fast movement of the US probe in combination. Furthermore, the change in US device from GE LOGIQ L9 (GE Healthcare, Chicago, IL, USA) to GE LOGIQ E9 in course of the study had a decisive impact on US image quality and made comparability more difficult during changeover period.
- The influence of the observer-dependency has not been investigated. The introduced SOP was created to minimize interobserver variability with respect to the image acquisition. However, this aspect has not been investigated in the current study and needs to be considered in future research approaches.

### Outlook

The precision of the UCL could be further improved in the future with advancements in medical technology, including US devices, structured reports and the implementation of artificial intelligence. The latter has already proved helpful in the assessment of thyroid nodules [41, 42]. The capacity to invalidate potentially suspicious findings represents a significant advantage of the UCL, as it can prevent the need for further diagnostics and invasive interventions. This could potentially reduce costs and save capacity, particularly in cases where invalidation through UCL occurs. Patients may also experience fewer side effects from radiation or radioactive drugs.

## Conclusions

In 12.6% of the patients, the additional acquisition and PACS archiving of cervical UCL revealed clinical relevance in the course of DTC disease. The invalidation of suspicious findings through the retrospective analysis of former UCL occurred as the most significant benefit of this method. The UCL SOP can be easily and quickly integrated into the US workflow.

## Abbreviations

CT: computed tomography
DTC: differentiated thyroid carcinoma
F-18-FDG: fluorine-18-fluorodeoxyglucose
FTC: follicular thyroid carcinoma
I-131: iodine-131
ID: initial diagnosis
MRI: magnetic resonance imaging
PACS: Picture Archiving and Communication System
PET: positron emission tomography
PTC: papillary thyroid carcinoma
PTMC: papillary thyroid carcinoma
RIS: Radiology Information System
RIT: radio iodine therapy
SOP: standard operating procedure
TG: thyroglobulin
(rh)TSH: (recombinant human) thyrotropin
TT: total thyroidectomy
UCL: standardized cervical ultrasound cine loops
UCL-CR: standardized cervical ultrasound cine loops with clinical relevance
UCL-CR^con^: confirmation of a suspicious finding identified on conventional live ultrasound
UCL-CR^ide^: identification of a suspicious finding that was not identified on conventional live ultrasound
UCL-CR^inv^: invalidation of a suspicious finding identified on conventional live ultrasound
UCL-nCR: standardized cervical ultrasound cine loop without clinical relevance
US: ultrasound
WBS: whole body scintigraphy
